# Positive Valence Bias in L2 Vocabulary Acquisition: Evidence From Chinese Emotion Idioms

**DOI:** 10.3389/fpsyg.2022.783604

**Published:** 2022-03-16

**Authors:** Mengxing Wang, Li Li, Jiushu Xie, Yaoyao Wang, Yao Chen, Ruiming Wang

**Affiliations:** ^1^School of Foreign Studies, South China Normal University, Guangzhou, China; ^2^The Key Laboratory of Chinese Learning and International Promotion, and College of International Culture, South China Normal University, Guangzhou, China; ^3^School of Psychology, Nanjing Normal University, Nanjing, China; ^4^Lijin Primary School, Shenzhen, China; ^5^Philosophy and Social Science Laboratory of Reading and Development in Children and Adolescents (South China Normal University), Ministry of Education, Guangzhou, China

**Keywords:** Chinese as a second language learners, positive valence bias, Chinese emotion idioms, semantic information, the L2 vocabulary acquisition model

## Abstract

Positive valence bias refers to speakers responding faster to positive than negative information in L2 emotion words. Few researchers paid attention to the initial learning phase of L2 Chinese emotion idioms in which whether positive valence bias was acquired, based on the three-stage model of L2 vocabulary acquisition. Besides, whether the semantic information would modulate positive valence bias at the initial learning phase remained unclear. This study reports two experiments on speakers learning Chinese as a second language (CSL) to investigate positive valence bias in the initial learning phase of new Chinese emotion idioms and the modulation of semantic information on positive valence bias. Chinese as a second language speakers, who had acquired new Chinese emotion idioms and passed the test for learned Chinese idioms with a high accuracy rate before formal experiments, participated in Experiments 1 and 2. In Experiment 1, target materials were new Chinese idioms with positive and negative information. Positive valence bias at the initial learning phase of Chinese idioms was investigated with valence judgments. Experiment 2 used a semantic relatedness decision task further to explore the semantic effect on positive valence bias. The result in the first experiment showed that positive valence bias appeared in Chinese emotion idioms even at the initial learning phase of the acquisition. Meanwhile, semantic information of Chinese emotion idioms appeared to affect positive valence bias in the infant learning phase in Experiment 2. The findings revealed that semantic information would affect the performance of positive valence bias, suggesting that the semantic processing would automatically access the valence at the infant learning phase L2 Chinese emotion idioms. The research results provided evidence that positive valence bias would form in the infant learning phase of Chinese emotion idiom acquisition, based on the L2 vocabulary acquisition model.

## Introduction

People prefer to listen to positive words in daily communication. Psycholinguistic studies on emotion words provided evidence to support this preference in language. Researchers found that positive words have a speed advantage over negative ones in the processing of emotion words with high arousal and either high (positive) or low (negative) valence (Pavlenko, 2008). Specifically, participants could make faster responses to positive words compared with negative counterparts, and this phenomenon was called positive valence bias (Dodds et al., 2015; Wang et al., 2019). This positive preference has been confirmed in a second language as well. Eilola and Havelka (2010) invited native English speakers and English as a second language (L2) speakers to participate in behavioral experiments with emotional Stroop tasks. The result showed that the two groups of participants both made slower responses to negative words significantly, in contrast to positive ones, suggesting that positive valence bias also appeared in the L2 emotion words. This finding had been proved in other related L2 studies on emotion words (Eilola et al., 2007; Sutton et al., 2007; Altarriba and Basnight-Brown, 2010; Degner et al., 2012).

However, whether this positive valence bias in the L2 emotion words could be generalized into the L2 Chinese emotion idioms with different features from emotion words remained unknown. Chinese emotion idioms, to some extent, differ from common emotion words on the number of characters and meanings. In general, compared with four-character Chinese emotion idioms, the most common emotion words consist of less than four characters in Chinese (e.g., “开心”/kāi xīn/in Pinyin, meaning to be happy in English, “难过” /nán guò/in Pinyin, meaning to be sad in English). With the feature of metaphor, Chinese emotion idioms include literal and figurative meanings, while the common emotion words only have a literal meaning. In other words, Chinese emotion idioms describe people’s feelings and emotions by metaphorical connotations, not the literal meaning. Wang et al. (2015) and Yu (2018) while common emotion words only bear the literal meaning to convey people’s thoughts. Despite some differences between Chinese emotion idioms and emotion words, speakers learning Chinese as a second language (CSL) all have to experience a similar gradual learning process to grasp and apply L2 Chinese idioms and words flexibly, especially for the late L2 learners.

According to the model of L2 vocabulary acquisition proposed by Jiang (2000), the late L2 speakers were obliged to experience three stages to reach the flexible application of L2 vocabulary. The first one is called the formal stage, where phonological and orthographic forms of a new word enter into a lexical entry. Next is the first language (L1) lemma mediation stage. In this stage, the lemma information, including semantic information of a new L2 word, is absorbed into the lexical entry and regulates L2 word usage by its L1 translation equivalents. The last stage is the L2 integration stage. This stage incorporates semantic, syntactic, and morphological information into the lexical entry when the new word is used frequently in real contexts. Based on the three stages in the L2 vocabulary acquisition model, speakers could learn the L2 vocabulary’s forms, semantics, and usage of L1 translation equivalents in the first two stages, classified as the initial learning phase in general, and the last one mainly referred to the application in natural contexts called the subsequent application phase by and large. In other words, late L2 speakers acquired L2 emotion words gradually by the initial learning phase and next application phase. Most studies in the second language showed that positive valence bias appeared in L2 emotion words people had developed and applied proficiently (Eilola et al., 2007; Sutton et al., 2007; Altarriba and Basnight-Brown, 2010; Degner et al., 2012). Few studies focused on this positive bias in the initial learning phase of L2 vocabulary acquisition. Thus, it seemed little known on the appearance of this positive bias in the initial learning phase of L2 vocabulary acquisition.

For emotion words with semantics and emotion information, there were two different accounts on the relation between semantics and emotion information. One assumption suggested that semantic information processing was independent of the emotion information, showing that semantic processing varied independently from emotion information (Opitz and Degner, 2012; Chen et al., 2015). In contrast, the other account suggested that semantic information processing was strongly associated with emotional information (Storbeck and Robinson, 2004; Eilola and Havelka, 2010; Dudschig et al., 2014; Sianipar et al., 2015). One behavioral study revealed that bilinguals processed the L2 emotion information depending on the access to the semantics (Storbeck and Robinson, 2004). Then, Eilola and Havelka (2010) proposed that the semantic processing of emotion words could refer to the automatic access to valence (Pavlenko, 2012). Or rather, the semantics of emotion words have a closer connection with valence rather than other factors among affective information. Much less research, however, has explored whether semantic processing was involved in the automatic access to valence. Besides, Eilola and Havelka (2010) focused on the familiar emotion words in which semantic processing automatically activated the valence. In other words, whether semantic processing in the initial learning phase of emotion words is directly accessible to the valence seemed unclear. If so, the semantic modulation of the valence processing should be demonstrated in the study.

As mentioned earlier, our study would pay more attention to three research questions. One was about whether positive valence bias would appear in L2 Chinese emotion idioms since a gradual learning process of L2 Chinese idioms is similar to L2 vocabulary. The second one was concerned with positive valence bias in the initial learning phase of L2 vocabulary. Another one referred to whether semantic processing of L2 Chinese emotion idioms would have access to the valence in the infant learning phase. Therefore, this study would design two experiments to explore three research questions mentioned earlier. Our main goal was to test further the appearance of positive valence bias in the initial learning phase of L2 Chinese emotion idiom acquisition in Experiment 1 by integrating the first two research questions. Experiment 2 would concentrate on the semantic effect on valence at the initial learning phase of L2 Chinese emotion idiom acquisition by manipulating the semantic factor of Chinese emotion idioms.

## Materials and Methods

### Experiment 1

#### Participants

Thirty-four junior CSL learners (8 male, 20–27 years old, and mean age = 22.7 years) from the School of International Culture, South China Normal University, were recruited for the following two experiments. They were right-handed with normal or corrected normal vision. Regarding their native language, 24 learners were native Indonesian speakers, and the remaining ten learners were native Thai speakers. As for L2 language proficiency, twenty participants have passed HSK 4 (“Hanyu Shuiping Kaoshi,” an international standardized test of Chinese language proficiency, with levels from 1 representing the ability to communicate in basic Mandarin to 6 representing to be proficient as a native speaker). The rest of the participants succeeded in HSK5 without taking part in HSK4. Generally speaking, CSL international students could basically reach the level of HSK 4 at the end of sophomore year. Passing HSK4 or even HSK5 means, CSL learners were able to discuss complicated topics in paragraphs, such as Chinese culture and cross-cultural topics, reaching the (upper) intermediate HSK levels (The Office of Chinese Language Council International, 2007). They all completed a language background and proficiency questionnaire before the experiment. The mean age of the Chinese acquisition (AoA) was 14.56 (±6.03) years. Their self-rated levels of Mandarin in speaking (*M* = 5.09 and SD = 0.82), listening (*M* = 4.97 and SD = 0.82), reading (*M* = 4.90 and SD = 1.12), and writing (*M* = 4.35 and SD = 1.28), from 1 (very bad) to 7 (very good), were calculated.

#### Materials

Ninety-six Chinese emotion idioms with four characters (including 48 positive idioms and 48 negative idioms) were first chosen from the Complete Dictionary of Chinese Idioms (Wang, 2008) and the Chinese Idiom Dictionary (Yu and Sun, 2004). Then, 47 native Chinese college students from South China Normal University rated the valence of chosen idioms from 1 (very negative) to 7 (very positive). The scorer reliabilities for 47 native raters were calculated using the Kendall W coefficient of concordance (Siegel, 1956), showing a good inter-rater agreement (the Kendall W coefficient of concordance = 0.68 and *p* = 0.00). The frequency and the total number of strokes of these idioms were computed with BCC Chinese corpus (Beijing Language and Culture University-Corpus Center) (Xun et al., 2016). A total of 30 Chinese idioms (half were higher than the mean valence of all 48 positive idioms and the other half were lower than the mean valence of all 48 negative idioms) were selected, and these 30 selected idioms were not taught in class according to the feedback of the teachers for teaching Chinese as a Second Language (TCSL) from the School of International Culture, South China Normal University. Meanwhile, eight CSL juniors who did not participate in the formal experiment were invited to pick out the idioms with which they were unfamiliar. Specifically, the eight CSL juniors were asked to explain the meanings of 30 idioms and make a sentence with each idiom. The meanings and sentences with idioms they provided were evaluated by TCSL teachers. The 24 idioms were picked out on the basis of the wrong meanings and incorrect sentences these CSL students gave. In total, 24 Chinese emotion idioms (half were positive and the other half were negative) were selected from 30 idioms. All the Chinese characters in the selected idioms were familiar in form but strange in semantics to the participants in the experiments. Twenty-four selected Chinese emotion idioms were target materials in the first experiment. The characteristics of all Chinese emotion idioms are presented in Table 1, and 24 selected Chinese emotion idioms are listed in Table 2. The rating revealed a significant difference in valence between positive and negative Chinese emotion idioms (*t*_11_ = 7.58 and *p* < 0.001). But both positive and negative idioms were similar in frequency (*t*_11_ = 0.01 and *p* = 0.99) and strokes (*t*_11_ = 0.44 and *p* = 0.67).

**TABLE 1:** Characteristics of Chinese emotion idiom in the experiments.

Condition	Valence	Frequency	Strokes
Positive idiom	5.67(0.16)	1520(973.53)	33.33(6.18)
Negative idiom	2.37(0.12)	1259.3(650.91)	32.16(7.47)

**TABLE 2:** Twenty-four Chinese emotion idioms and their Pinyin.

No.	Chinese idioms	Pinyin	English translation
			
1	喜气洋洋	/xǐ qì yáng yáng/	To be bursting with happiness
2	兴高采烈	/xìng gāo cǎi liè/	To be in good spirits
3	春风得意	/chūn fēng dé yì/	To be extremely proud of one’s success
4	心满意足	/xīn mǎn yì zú/	To be fully satisfied and content
5	神采奕奕	/shén cǎi yì yì/	To be in good out of a bandbox
6	欣喜若狂	/xīn xǐ ruò kuáng/	To be wild with joy
7	手舞足蹈	/shǒu wǔ zú dǎo/	To dance with joy
8	满面春风	/mǎn miàn chūn fēng/	To shine with happiness
9	欢天喜地	/huān tiān xǐ dì/	To be elated and happy
10	眉飞色舞	/méi fēi sè wǔ/	To beam with joy
11	大快人心	/dà kuài rén xīn/	This cheers the people greatly
12	皆大欢喜	/jiē dà huān xǐ/	To the satisfaction of all
13	火冒三丈	/huǒ mào sān zhàng/	To fly into a rage
14	惊慌失措	/ j īng huāng shī cuò/	To be panic-stricken
15	心乱如麻	/xīn luàn rú má/	To be utterly upset
16	心急如焚	/xīn jí rú fén/	One’s heart is torn with anxiety
17	嚎啕大哭	/háo táo dà kū/	To cry bitter tears
18	怒发冲冠	/nù fà chōng guān/	To bristle with anger
19	勃然大怒	/bó rán dà nù/	To burst into anger
20	暴跳如雷	/bào tiào rú léi/	To stamp with fury
21	毛骨悚然	/máo gǔ sǒng rán/	To be thrilling
22	惴惴不安	/zhuì zhuì bù ān/	To be anxious and fearful
23	怒气冲天	/nù qì chōng tiān/	To be furious
24	怒火中烧	/nù huǒ zhōng shāo/	To simmer with rage

*This table demonstrates 12 positive idioms from No. 1 to 12 and 12 negative idioms from No. 13 to 24 with Pinyin and English translation.*

#### Design and Procedure

We used a single factor (types of valence: positive idiom and negative idiom) within-subject design for exploration. The dependent variables were reaction times (RTs) and accuracy (ACC).

**FIGURE 1:**
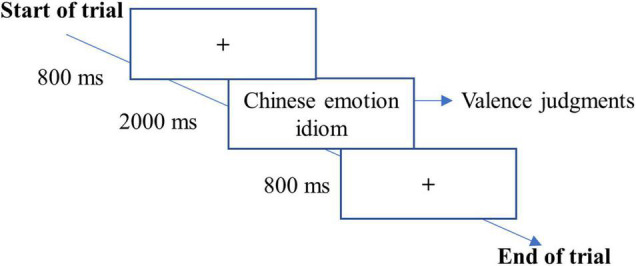
A trial used in Experiment 1.

Before the formal experiments, 34 participants were taught 24 selected Chinese emotion idioms for 80 min in class with annotations, examples, and pictures. After learning, CSL learners had to complete a test by reading Chinese emotion idioms and choosing the right one to finish a sentence. At last, all participants grasped these Chinese emotion idioms to reach a 90% ACC rate in this test. Then, 34 participants who passed the test took part in the first experiment. The task of the experiment mainly asked participants to make valence judgments on newly learned Chinese emotion idioms presented at the center of the screen. The task was conducted using E-prime 2.0.10. In total, there were 24 trials. Each trial of the task began with the presentation of fixation cross “+” for 800 ms. Then, a Chinese emotion idiom was followed, where participants were required to judge the valence of emotion idioms. To be specific, they had to judge whether the idiom was positive or negative (by pressing the “F” or “J” key). One-half participants pressed the “F” key when seeing the positive idioms and pressed the “J” key for negative stimuli, while the other half responded to the positive idioms with the “J” key and chose the “F” key responding to negative ones. The presented Chinese emotion idiom would remain on the screen until a judgment was given or after 2,000 ms had passed. Before the formal experiment, there were four practice trials (Figure 1).

#### Results

Three participants were removed from the analysis, given that their ACC rates were lower than 50%. Besides, the error trials were excluded, and RTs in the correct trials beyond 2.5 SDs from the mean were also removed as outliers. Based on the two removal criteria mentioned earlier, 19% of data were deleted. The descriptive results are shown in Table 3.

**TABLE 3:** Mean reaction times (RTs) and accuracy (ACC) in Experiment 1 (SDs in parentheses).

Type of valence	Positive idiom	Negative idiom
RTs	961(326)	1077(352)
ACC	0.84(0.13)	0.79(0.17)
		

Reaction time analysis was conducted with mixed-effect models, using the lme4 package (Bates et al., 2007) and the lmerTest package (Kuznetsova et al., 2014) of the statistical software R 3.4.3. Mixed-effect models were used because the method took random effects of participants and items into consideration, offering a more appropriate way for us to model the data. This could generalize the results of this study to the other studies with similar subjects and items.

For RT analysis, we fitted a mixed-effect model with the type of valence (positive idiom vs. negative idiom) as a fixed effect. In addition, we included by-participants and by-item random intercepts as random effects. To determine the best-fitting structure in the study, we employed the forward comparison method (Bates et al., 2015; Matuschek et al., 2017). Table 4 gives a summary of results from the model for RTs. As shown in Tables 2, 4, we could find a significant difference in RTs between positive and negative idioms (*p* = 0.036) (Table 4), suggesting faster RT in positive idioms (961 ms) than in negative idioms (1,077 ms).

**TABLE 4:** Model parameters for the best-fitting model for RTs in Experiment 1.

Fixed effects	Estimate	SE	t	*p*
Intercept	6.82	0.05	135.34	< .001***
Type of valence	0.10	0.05	2.24	0.036 *

*p < 0.05; ***p < 0.001.

For ACC analysis, a generalized linear mixed-effect model with binomial distribution was used to analyze the ACC rate with the type of valence (positive idiom vs. negative idiom) as a fixed effect. By-participant and by-item random intercepts were included as random effects. Table 5 summarizes the results from the model for ACC. As shown in Tables 3, 5, the difference for the type of valence was not significant (*p* = 0.195). In general, the results showed that positive valence bias occurred in Chinese emotion idiom for RTs, and this phenomenon in RTs was not at the cost of reducing ACC.

**TABLE 5:** Model parameters for the best-fitting model for ACC in Experiment 1.

Fixed effects	Estimate	SE	z	*p*
Intercept	1.97	0.27	7.17	< 0.001***
Type of valence	–0.43	0.33	–1.30	0.195

***p < 0.001.

In brief, positive valence bias in emotion words was found in Chinese emotion idioms for CSL learners as well. Furthermore, this phenomenon in emotion words had been acquired well in the infant learning phase of L2 Chinese emotion idioms.

In emotion words, the semantics seemed to be associated with the valence. Eilola and Havelka (2010) found that semantics could directly access the valence of the emotion words (Pavlenko, 2012). Based on the L2 vocabulary acquisition model proposed by Jiang (2000), at the initial learning phase, semantic information of emotion idioms has been acquired. If this was the case, semantic information processing should have activated the valence in the infant learning phase of emotion idioms, suggesting the semantic modulation of the positive valence bias. In the second experiment, we, therefore, decided to investigate the semantic effect on positive valence bias in the infant learning phase of L2 Chinese emotion idiom acquisition by a semantic relatedness decision task applied in many psychological pieces of research (Silveri et al., 1996; Balota and Black, 1997; Zwaan and Yaxley, 2003). According to the assumption that semantic processing would be the access to valence, we could predict that the positivity bias only happens in the related semantics, revealing semantic processing would automatically activate valence in the initial phase of emotion idioms. Besides, with our attention to the initial learning phase of L2 Chinese emotion idioms, we invited the same participants to participate in Experiment 2.

### Experiment 2

#### Participants

The same thirty-four CSL learners in Experiment 1 participated in Experiment 2.

#### Materials

Prime words related and unrelated to target idioms in semantics were selected. Six related prime words in semantics for each Chinese emotion idiom were chosen from the BCC Chinese corpus (Beijing Language and Culture University-Corpus Center) (Xun et al., 2016). Then, the most related one in semantics from six prime words was selected with 289 online questionnaires. Moreover, specific words (e.g., “书本” /shū běn/ in Pinyin, meaning “book” in English) were chosen as unrelated prime words. These related and unrelated words are listed in Table 6. Fifteen native Chinese speakers were invited to rate the semantic relatedness between the prime words (related and unrelated) and emotion idioms (positive and negative), using a seven-point scale (1 = very unrelated and 7 = very related). The scorer reliabilities for 15 native Chinese raters were calculated using the Kendall W coefficient of concordance (Siegel, 1956), showing a good inter-rater agreement (the Kendall W coefficient of concordance = 0.725 and *p* = 0.00). The rating data of the semantic relatedness between emotion idioms (positive and negative idioms) and prime words (related and unrelated) was analyzed using a two-way ANOVA. The results showed no significant main effect of emotion idioms (positive and negative idioms) on the semantic relatedness, *F*(1, 11) = 0.948 and *p* = 0.35, while there was a significant main effect of prime words (related and unrelated) on the semantic relatedness, *F*(1,11) = 2,216.12 and *p* < 0.001, suggesting the distinct difference on the semantic relatedness between the related and unrelated prime words on the semantics. Besides, the interaction of the semantic relatedness between emotion idioms and prime words was also insignificant, *F*(1,11) = 1.539 and *p* = 0.24. The frequency and the total number of strokes of prime words were computed with BCC Chinese corpus (Beijing Language and Culture University-Corpus Center) (Xun et al., 2016). These characteristics of prime words are listed in Table 7. Both prime words in positive and negative idioms were similar in frequency (*t*_46_ = 1.38 and *p* = 0.18) and strokes (*t*_46_ = -0.39 and *p* = 0.70). Fillers contained six positives and six negative four-character words (e.g., “开开心心”/kāi kāi xīn xīn/ in Pinyin, meaning to be happy in English; “十分生气”/shí fēn shēng qì/ in Pinyin, meaning to be very angry in English), not idioms. Before Experiment 2, TCSL teachers from the School of International Culture, South China Normal University, were invited to revise all the prime words and fillers to ensure that junior CSL speakers had learned these words in class. Besides, eight CSL juniors involved in selecting the target idioms could also recognize and tell the meanings of these prime words and fillers without participating in the formal experiments.

**TABLE 6:** Twenty-four Chinese emotion idioms, and the related and unrelated words.

No.	Chinese idioms	Related word, Pinyin and English translation	Unrelated word, Pinyin and English translation
			
1	喜气洋洋	新年/xīn nián/ new year	键盘/jiàn pán/ keyboard
2	兴高采烈	礼物/lǐ wù/gift	纸巾/zhǐ j īn/ tissue
3	春风得意	事业/shì yè/ business	闹钟/nào zhōng/alarm clock
4	心满意足	成就/chéng jiù/ achievement	玻璃/bō lí/ glass
5	神采奕奕	精神/jīng shén/ spirit	日历/rì lì/ calendar
6	欣喜若狂	优胜/yōu shèng/ winning	土地/tǔ dì/ land
7	手舞足蹈	胜利/shèng lì/ victory	窗帘/chuāng lián/ curtain
8	满面春风	赞扬/zàn yáng/ praise	沙漠/shā mò/ desert
9	欢天喜地	孩子/hái zi/ children	厕所/cè suǒ/ toilet
10	眉飞色舞	趣闻/qù wén / anecdotes	书本/shū běn/ book
11	大快人心	惩处/chéng chǔ/ punishment	手表/shǒu biǎo/ watch
12	皆大欢喜	结局/jié jú/ ending	铅笔/qiān bǐ/ pencil
13	火冒三丈	拳头/quán tóu/ fist	胶带/jiāo dài/ sticky tape
14	惊慌失措	火灾/huǒ zāi/ fire	苹果/píng guǒ/ apple
15	心乱如麻	矛盾/máo dùn/ contradict	白云/bái yún/ cloud
16	心急如焚	意外/yì wài/ accident	橡皮/xiàng pí/ rubber
17	嚎啕大哭	哭声/kū shēng/ cry	白菜/bái cài/ cabbage
18	怒发冲冠	怒气/nù qì/ anger	鲜花/xiān huā/ flowers
19	勃然大怒	混账/hùn zhàng/scoundrel	火车/huǒ chē/ train
20	暴跳如雷	脾气/pí qi/ temper	树叶/shù yè/ leaf
21	毛骨悚然	鬼怪/guǐ guài/ monster	面包/miàn bāo/ bread
22	惴惴不安	局势/jú shì/ situation	衣服/yī fú/ clothes
23	怒气冲天	恶棍/è gùn/ villain	梦想/mèng xiǎng/ dream
24	怒火中烧	激愤/jī fèn/ Indignant	冰箱/bīng xiāng/ fridge

*This table demonstrates 12 positive idioms from No. 1 to 12 and 12 negative idioms from No. 13 to 24 with their related and unrelated prime words.*

**TABLE 7:** Characteristics of prime words in Experiment 2.

Condition	Semantic relatedness	Frequency	Strokes
Related prime words in positive idioms	6.04(0.34)	40106(58132)	17.33(3.84)
in negative idioms	6.07(0.58)	10488(10074)	17.08(4.29)
Unrelated prime words in positive idioms	1.35(0.30)	8092(13258)	15.00(5.75)
in negative idioms	1.13(0.10)	11524(16923)	16.33(4.89)

*This table demonstrates the semantic relatedness between the prime words and emotion idioms, the corresponding frequency, and strokes of prime words.*

#### Design and Procedure

We used 2 (types of valence: positive idiom and negative idiom)×2 (semantic relatedness: related and unrelated) within-subject design in Experiment 2, with RTs and ACC as dependent variables.

**FIGURE 2:**
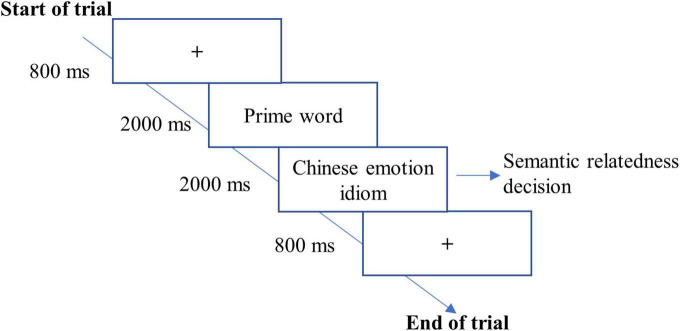
A trial used in Experiment 2.

This study conducts a semantic relatedness decision task to determine whether semantic information would modulate positive valence bias in the L2 Chinese emotion idiom learning phase. There were 60 trials in total for this experiment. Each trial began with the presentation of fixation cross “+” for 800 ms, followed by a prime word for 2,000 ms. Then, the target Chinese emotion idiom was displayed for 2,000 ms or disappeared when a response was given. Participants were instructed to decide whether the target Chinese emotion idiom was related to the prime word in semantics. They responded to this task as quickly and accurately as possible by pressing the “F” or “J” key, which were counterbalanced across participants. Before the experimental session, participants first completed four practice trials (Figure 2).

#### Results

Five participants whose ACC rate was below 50% were removed from data analysis. The rest of the data removal procedures were identical to Experiment 1. Then, 72% of the data were kept for further analysis. Descriptive results are shown in Table 8.

**TABLE 8:** Mean RTs and ACC in Experiment 2 (SDs in parentheses).

Type of valence	Positive idiom	Negative idiom
**RTs**		
Related	1034(333)	1168(339)
Unrelated	1162(357)	1149(353)
**ACC**		
Related	0.75(0.15)	0.64(0.19)
Unrelated	0.72(0.24)	0.80(0.14)

We fitted a mixed-effect model for RT analyses with the type of valence (positive idiom vs. negative idiom), semantic relatedness (related vs. unrelated), and their interactions as fixed effects. Prime word frequency, word frequency of idiom, language background (AoA, the first language), L2 language proficiency (listening, speaking, reading, and writing) were added into this mixed-effect model as covariates. We considered by-participant and by-item random intercepts and by-participant random slopes for the valence and semantic relatedness and their interaction as random effects. The forward comparison method was employed to determine the best-fitting structure in the study (Bates et al., 2015; Matuschek et al., 2017).

**FIGURE 3:**
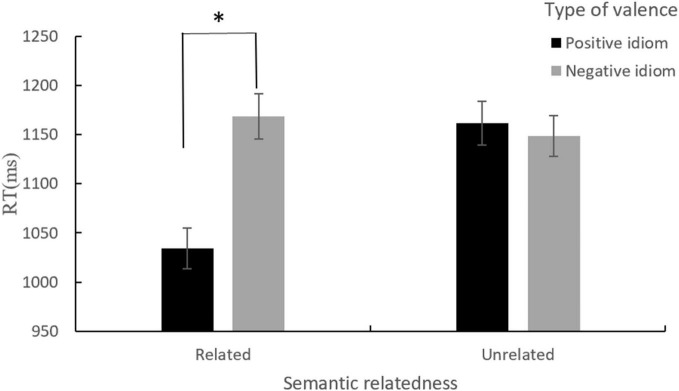
An interaction between semantic relatedness and type of valence for RTs. *p < 0.05.

As shown in Figure 3 and Tables 8, 9, no main effects were significant but the interaction between type of valence and semantic relatedness was significant (*p* = 0.023). To further understand this interaction, we conducted separate sub-models for related semantics and unrelated semantics. In the related semantics, we found that the main effect of valence was significant (*p* = 0.018), suggesting the appearance of positive valence bias. In the unrelated semantics, the main effect of valence was nonsignificant (*p* = 0.466), indicating no positive valence bias. Among the covariates, only the idiom frequency was significant (*p* = 0.047), and other covariates were insignificant (Table 9).

**TABLE 9:** Model parameters in the mixed-effect model for RTs in Experiment 2.

Fixed effects	Estimate	SE	*t*	*p*
Intercept	7.38	0.19	38.30	< 0.001***
Type of valence	0.04	0.03	1.36	0.18
Semantic Relatedness	0.05	0.04	1.44	0.16
Prime word frequency	0.24	0.02	1.43	0.16
Word frequency of idiom	–0.03	0.01	–2.05	0.047*
Listening	–0.04	0.05	–0.90	0.38
Spoken	0.02	0.05	0.43	0.67
Reading	–0.01	0.04	–0.18	0.86
Writing	–0.07	0.04	–1.83	0.08
AoA	0.01	0.00	1.28	0.21
First language	0.06	0.07	0.88	0.39
Type of valence **×** Semantic Relatedness	–0.15	0.06	–2.36	0.02*

*p < 0.05; ***p < 0.001.

We conducted a generalized linear mixed-effect model with a binomial distribution for ACC analyses, with the same fixed effect structure, covariates, and random effect structure as the linear mixed-effect model for RTs. For this model, the variables were coded using a mean-centered contrast. However, the two main effects and interaction effects were not statistically significant (Table 10). These results showed that this positive valence bias shown in RTs was not at the expense of reducing ACC.

**TABLE 10:** Model parameters in the mixed-effect model for ACC in Experiment 2.

Fixed effects	Estimate	SE	*z*	*p*
Intercept	1.19	0.14	8.28	< 0.001***
Type of valence	–0.09	0.22	–0.40	0.69
Semantic Relatedness	0.54	0.31	1.75	0.08
Prime word frequency	0.02	0.02	1.43	0.16
Word frequency of idiom	0.04	0.10	0.37	0.71
Listening	0.09	0.18	0.52	0.61
Spoken	0.12	0.15	0.81	0.42
Reading	–0.04	0.13	–0.33	0.74
Writing	0.18	0.14	1.32	0.19
AoA	–0.01	0.01	–0.76	0.45
First language	–0.37	0.22	–1.71	0.09
Type of valence × Semantic Relatedness	0.91	0.45	2.04	0.076

***p < 0.001.

In summary, the main effect of valence was significant in the related semantics, while no significant signs were in the unrelated semantics, revealing that in the initial learning phase of L2 Chinese emotion idiom acquisition, the semantic information would conditionally modulate the positive valence bias. This finding consistent with our prediction has confirmed the automatic access to the valence in the semantic information processing.

## Discussion

This study mainly investigates positive valence bias and semantic modulation on this bias in the initial learning phase of L2 Chinese emotion idiom acquisition. In Experiment 1, immediately after CSL participants had learned Chinese emotion idioms, positive valence bias in Chinese idioms was found. This result suggested that positive valence bias could occur even at the initial learning phase of L2 Chinese emotion idioms. In Experiment 2, newly learned positive valence bias was measured with semantic relatedness decision task. The significant result revealed that the semantics seemed to conditionally modulate positive valence bias in the earlier learning phase of L2 Chinese emotion idioms. Overall, one remarkable finding was revealed that positive valence bias presented not only in L2 Chinese emotion idioms but also at the earlier learning phase. The other significant finding showed that in the initial learning phase of Chinese emotion idioms, the semantic information still would modulate the presence of positive valence bias, suggesting that semantic processing would be involved in the access to valence. Then, we would illustrate the results of this study from two aspects, positive valence bias and its semantic factor.

Positive valence bias was also found in L2 Chinese emotion idioms. This finding was in line with previous studies on L2 emotion words (Eilola et al., 2007; Sutton et al., 2007; Altarriba and Basnight-Brown, 2010; Eilola and Havelka, 2010; Degner et al., 2012). Eilola and Havelka (2010) used the common emotion words with which L2 speakers were familiar and discovered positive valence bias in L2 emotion words. In other words, positive valence bias in L2 emotion words could be generalized to L2 Chinese emotion idioms, based on the finding in our study.

Furthermore, positive valence bias occurring in L2 familiar emotion words had formed very early since this valence bias appeared in the initial learning phase of L2 Chinese idiom acquisition. According to the three-stage model of L2 vocabulary acquisition put forward by Jiang (2000), we supposed that CSL learners might have experienced three similar stages in Chinese idiom acquisition, just as L2 vocabulary. At the first stage, learners are familiar with the formal information of Chinese idioms. At the second stage, they should independently develop and grasp new semantics and lemma in the idiom learning without bypassing L1 translation equivalents. Finally, CSL learners could use Chinese idioms proficiently in the context. Based on this finding in our study, positive valence bias seemed to form in the first two stages of the L2 acquisition model rather than after the final application stage. In other words, positive valence bias could be possible to present in the initial learning phase of L2 emotion vocabulary acquisition, which is consistent with the observation from most L2 emotion words in other studies that people have acquired and applied proficiently (Eilola et al., 2007; Sutton et al., 2007; Altarriba and Basnight-Brown, 2010; Eilola and Havelka, 2010; Degner et al., 2012). Besides, the initial learning phase included the first two stages based on L2 vocabulary acquisition (Jiang, 2000). Specifically, which one in the first two stages of L2 vocabulary acquisition would be closer relevant to the appearance of positive valence bias remained further to be explored in the future.

In Experiment 2, CSL participants showed faster responses to positive idioms than negative ones in processing related semantic information. This significant finding in semantic factor proved that this positivity bias was well-established in the initial learning phase of emotion idioms again since positive valence bias in the valence judgment task also appeared in the semantic relatedness decision task. Besides, the result that semantic effect on positive valence bias verified that semantic processing did get automatic access to valence information in emotion words (Eilola and Havelka, 2010; Pavlenko, 2012). Positivity bias happened in the related semantic condition but not in the unrelated semantics, suggesting that the processing of related semantics facilitated the access to valence, and the semantic unrelatedness did not activate the valence information, resulting in the disappearance of positive valence bias.

More generally, our result that semantic modulation of the positivity bias provided further evidence for the assumption that the processing of semantic information was associated with the emotion information, especially with the valence in emotion words. Furthermore, the clear demonstration of semantic effect on positive valence bias suggested that, at least, at the initial learning phase of emotion idioms, semantic processing is connected with emotion information rather than independent of the emotion information.

Word frequency as a factor would influence the speed for responding to familiar emotion words with different valence (Chastain et al., 1996; Burt, 2002; Kahan and Hely, 2008). Burt (2002) found that participants responded faster to familiar emotion words with high word frequency in the emotional Stroop task than low-frequency emotion words in the experiments. Based on these studies, word frequency as a covariate in this study was controlled as the frequency was unrelated to our research purposes. We found that the semantic modulation of positive valence bias could be observed when the influence from word frequency of idiom was removed as a covariate. It suggested that word frequency of idioms still affects response speed in the initial learning phase of Chinese emotion idioms. Thus, word frequency of idioms seemingly was worth investigating as a variable at the earlier learning phase of emotion words in the future.

Interestingly, regarding the ACC in the two experiments, there were different performances between the two tasks, although the ACC rate of all conditions from the two tasks was higher than the chance level. The ACC rate was nearly higher than 80% in the valence judgment task, while the ACC rate of the semantic relatedness judgment task was lower than 80%. Based on the discrepancy in ACC for the processing of valence and semantics, we assumed that CSL learners were more prone to grasp and judge the valence of Chinese emotion idiom acquisition than semantics. In addition, with the two experiments’ distinct research proposes, the difference in task difficulty was plausibly responsible for the discrepancy in the ACC. Specifically, compared with the valence task referring to target idioms, the semantic relatedness task was possibly more complicated, involving the semantic association between prime words and target idioms.

There were some limitations in the research. This one limitation was about the learning method in the learning phase. Participants were required to learn 24 Chinese emotion idioms for 80 min in class at a time. This approach to learning would enable participants to feel fatigued. However, this tiredness had the same effect on the positive and negative idioms for idioms in a balanced way. The other referred to the language background of participants. With the high requirement for L2 language proficiency, we could not find enough CSL learners with HSK 4 from the same country. Therefore, participants were mainly from Indonesia and Thai. Some CSL participants were at the level of HSK 4, but a few were with HSK 5. The difference in the L1 background and L2 proficiency of CSL learners might have less influence on Chinese emotion idioms acquisition. Regarding the possible influence of language background and L2 language proficiency on the results, the two factors were controlled as covariates in the statistical analysis. The results showed that language background and language proficiency seemingly fail to affect the performance of positive valence bias.

In the future, it might be interesting to investigate further the stages in a model where positive valence bias would be acquired, e.g., which stage at the initial learning phase of Chinese emotion idioms is closer related to the presence of positive valence bias.

## Conclusion

This study found evidence that CSL learners instantly responded faster to positive information than negative information after learning new Chinese emotion idioms, supporting that in the initial learning phase of Chinese emotion idiom acquisition, positive valence bias had been learned. Furthermore, we observed the positive valence bias in the semantically related condition, but not in the semantically unrelated condition, indicating that the semantic information would conditionally regulate this positive valence bias in this learning phase, at least for L2 Chinese emotion idiom acquisition. Again, the results supported positive valence bias acquisition in the initial learning phase of Chinese emotion idioms and even verified that semantic processing was direct to access valence, not independent of emotion information. Our findings showed that positive valence bias did form in the infant learning phase of L2 Chinese emotion idioms, discovered the processing of semantics accessible to valence, and supported the assumption that semantics was associated with the emotion information.

## Data Availability Statement

The original contributions presented in the study are included in the article/supplementary material, further inquiries can be directed to the corresponding authors.

## Ethics Statement

The studies involving human participants were reviewed and approved by Human Research Ethics Committee for Non-Clinical Faculties; The School of Psychology, South China Normal University. The patients/participants provided their written informed consent to participate in this study.

## Author Contributions

LL and RW designed the experiments. YW ran the data-collection procedures of the experiments. MW and RW analyzed and interpreted the data. MW drafted the manuscript. LL, JX, and YC provided critical revisions of the manuscript. All authors contributed to the article and approved the submitted version.

## Conflict of Interest

The authors declare that the research was conducted in the absence of any commercial or financial relationships that could be construed as a potential conflict of interest.

## Publisher’s Note

All claims expressed in this article are solely those of the authors and do not necessarily represent those of their affiliated organizations, or those of the publisher, the editors and the reviewers. Any product that may be evaluated in this article, or claim that may be made by its manufacturer, is not guaranteed or endorsed by the publisher.
